# Avian Pox in Native Captive Psittacines, Brazil, 2015

**DOI:** 10.3201/eid2301.161133

**Published:** 2017-01

**Authors:** Felipe C.B. Esteves, Sandra Y. Marín, Maurício Resende, Aila S.G. Silva, Hannah L.G. Coelho, Mayara B. Barbosa, Natália S. D’Aparecida, José S. de Resende, Ana C.D. Torres, Nelson R.S. Martins

**Affiliations:** Universidade Federal de Minas Gerais, Belo Horizonte, Minas Gerais, Brazil

**Keywords:** Brazil, avian pox, psittacines, avipoxvirus, viruses, parrot, parakeet, macaw, birds

## Abstract

To investigate an outbreak of avian pox in psittacines in a conservation facility, we examined 94 birds of 10 psittacine species, including sick and healthy birds. We found psittacine pox virus in 23 of 27 sick birds and 4 of 67 healthy birds. Further characterization is needed for these isolates.

Avian pox is caused by avipoxvirus. Infections occur worldwide in domestic and wild avian species (*1*), are suggested to be host family- or order-specific, and are modulated by habitat and ecologic niche (*2*). Avipoxviruses have been described in Brazilian *Amazona* spp. and *Pionus* spp. parrots with severe diphtheritic upper digestive lesions, experimentally causing the formation of cutaneous lesions in chickens; chicken and parrot strains will not provide cross protection (*3*). The presumptive diagnosis, based on typical pocklike skin lesions of papular or nodular hyperplasic and hypertrophic skin foci or upper digestive diphtheritic form in severe cases (*1*), may be confirmed by detection of avipoxvirus DNA by PCR (*4*).

In June 2015, an outbreak of avian pox occurred among 10 species of native Brazilian psittacines (n = 94) maintained in a conservation facility. In addition to the typical pocklike nodular skin lesions, the psittacines had weight loss and reduced activity; 3 died. The outbreak lasted 3 months; remission of lesions occurred within ≈3 weeks in each bird.

Skin scrapings were collected from the cutaneous lesions of affected birds (Technical Appendix Figure 1), and conjunctiva and cloaca swabs were collected from all 94 psittacines showing cutaneous lesions (27 birds) or not (67 birds). Skin samples treated with an antibacterial–antimycotic drug mixture (Gibco; Thermo Fisher Scientific, Waltham, MA, USA) were inoculated onto the chorioallantoic membrane (CAM) of 10-day-old specific pathogen–free chicken embryos and typical pocklike CAM lesions were obtained (Technical Appendix Figure 2). Cutaneous samples and the CAM of inoculated embryos were subjected to PCR with specific primers forward 5′-CAGCAGGTGCTAAACAACAA-3′ and reverse 5′-CGGTAGCTTAACGCCGAATA-3′ (*4*) to amplify a partial sequence (576 bp) of the gene encoding the core protein P4b (*fpv167* locus) of avipoxvirus (Table). The avipoxvirus P4b gene partial sequence was obtained from skin lesions, conjunctiva and cloacal swabs, and CAM; sequences then underwent phylogenetic characterization (Technical Appendix Figure 3).

**Table:** Avipoxvirus detection by PCR according to clinical status and psittacine species, Brazil, 2015

Psittacine species	**Clinical status**				
	PCR positive			PCR negative	
	Normal	Cutaneous lesions		Normal	Cutaneous lesions
*Amazona aestiva *(blue-fronted parrot)	4	–		–	–
*Amazona brasiliensis *(red-tailed Amazon)	–	1		4	1
*Anodorhynchus hyacinthinus *(hyacinthine macaw)	–	2		4	–
*Ara chloropterus *(green-winged macaw)	–	–		2	–
*Ara macao *(scarlet macaw)	–	2		1	–
*Ara ararauna *(blue and yellow macaw)	–	–		14	–
*Deroptyus accipitrinus* (red-fan parrot)	–	1		9	2
*Guaruba guarouba *(golden conure)	–	9		15	1
*Pionites leucogaster *(white-bellied caique)	–	4		12	–
*Pionus fuscus *(dusky parrot)	–	3		2	–
Total	4	23		63	4
Ratio, no. birds/no. tested (%)	4/94 (4.2)	23/94 (24.4)		63/94 (67.0)	4/94 (4.2)

*Values are no. birds except as indicated. –, not found.

The P4b gene partial sequences obtained from the avipoxvirus isolate Betim-1 of the red-and-green macaw (*Ara chloropterus*) (GenBank accession no. KT187552), dusky parrot (*Pionus fuscus*) (Esteves et al., unpub. data), and golden conure (*Guaruba guarouba*) (Esteves et al., unpub. data) were 100% identical. The obtained sequences were aligned with local avipoxvirus of different species with clinical disease (Esteves et al., unpub. data) and with previously published avipoxvirus sequences in GenBank (Technical Appendix Figure 3). Evolutionary analyses were performed in MEGA6 (*5*), evolutionary history was inferred by neighbor joining (*6*), replicate trees were clustered by using the bootstrap test (*7*) with 1,000 oversampling, and evolutionary distances were computed by the Tamura 3-parameter method (*8*). The phylogenetic relationships revealed grouping with psittacine poxviruses, including isolates from the United Kingdom (macaw, GenBank accession no. AM050382.1, and parrot, GenBank accession no. AM050383.1); United States (yellow-crowned amazon [*Amazona ochrocephala*], GenBank accession no. KC018069.1); and Germany (lovebird [*Agapornis* sp.], GenBank accession no. AY530311.1). To enable comparisons, a local strain (GenBank accession no. KX863707) of the Atlantic canary (*Serinus canaria*) (Esteves et al., unpub. data) and an isolate of the Magellanic penguin (*Spheniscus magellanicus*) (GenBank accession no. KF516679.1) (*9*) were grouped in the canarypox clade. A local chicken isolate (GenBank accession no. KX863706) was grouped within the fowlpox clade, including chicken (GenBank accession nos. KF722860.1 KF722863.1) and turkey (GenBank accession nos. KF017961.1 and DQ873808.1) strains.

Birds with pocklike lesions represented 28.7% (27/94) of psittacines in the sanctuary. Laboratory diagnosis was implemented at the early stages of the outbreak; birds were clinically observed up to the final cases (3 months). Of the 27 psittacines with lesions, 23 (85.2%) were PCR positive for avipoxvirus. No blue-fronted parrots (*Amazona aestiva*) showed lesions, but all were PCR positive (4/94 [4.2%]), suggesting that they were immune, although they were not tested for an immune response. Among the 25 golden conures, the most abundant species in the premises, 9 showed typical pocklike lesions and were PCR positive, but 1 with lesions was PCR negative and 15 had no lesions and were PCR negative, indicating nonuniform immunity. None of the 14 blue and yellow macaws (*Ara ararauna*) had lesions; PCR results were negative for all, suggesting previous immunity or a lower susceptibility to infection. Four birds with lesions were not PCR positive, possibly indicating an advanced phase of virus-free scars. 

The proximity of aviaries suggests that all birds might have had the opportunity for infection during the outbreak. The death rate (3.2%) for the outbreak was low; the death of young golden conures with diphtheritic lesions and 2 adult dusky parrots that demonstrated conjunctivitis and cachexia were accompanied by complications by *Candida albicans* and *Capillaria* sp., suggesting the potential aggravation risk. Most psittacines (63/94; 67.0%) were PCR negative and without lesions, as tested in cloacal and conjunctival swabs.

The affected species are declining in wild populations. Our findings emphasize the risk for avipoxvirus among captive psittacines; their relevance for psittacine rehabilitation and conservation may be considerable regarding pathogenicity. As shown, 2 adult dusky parrots and 1 nestling golden conure died; further characterization is needed for the isolates, including their eventual importance for commercial poultry.

Technical Appendix Green-winged macaw with facial lesions, virus isolation in the chorioallantoic membrane with pocklike lesions, and the phylogenetic tree showing grouping of an isolate with *Psittacinepoxvirus*.

## References

[1] Tripathy DN, Reed WM. Pox. In: Saif YM, Barnes HJ, Glisson JR, Fadly AM, McDougald LR, Swayne DE, editors. Diseases of poultry. 11th ed. Ames (IA): Iowa State University Press; 2003. pp. 253–269.

[2] Gyuranecz M, Foster JT, Dán Á, Ip HS, Egstad KF, Parker PG, et al. Worldwide phylogenetic relationship of avian poxviruses. J Virol. 2013;87:4938–51. 10.1128/JVI.03183-12 23408635 PMC3624294

[3] Boosinger TR, Winterfield RW, Feldman DS, Dhillon AS. Psittacine pox virus: virus isolation and identification, transmission, and cross-challenge studies in parrots and chickens. Avian Dis. 1982;26:437–44. 10.2307/1590119 6285884

[4] Huw Lee L, Hwa Lee K. Application of the polymerase chain reaction for the diagnosis of fowl poxvirus infection. J Virol Methods. 1997;63:113–9. 10.1016/S0166-0934(96)02119-2 9015281

[5] Tamura K, Stecher G, Peterson D, Filipski A, Kumar S. MEGA6: Molecular Evolutionary Genetics Analysis version 6.0. Mol Biol Evol. 2013;30:2725–9. 10.1093/molbev/mst197 24132122 PMC3840312

[6] Saitou N, Nei M. The neighbor-joining method: a new method for reconstructing phylogenetic trees. Mol Biol Evol. 1987;4:406–25. 3447015 10.1093/oxfordjournals.molbev.a040454

[7] Felsenstein J. Confidence limits on phylogenies: an approach using the bootstrap. Evolution. 1985;39:783–91. 10.2307/2408678 28561359

[8] Tamura K. Estimation of the number of nucleotide substitutions when there are strong transition-transversion and G+C-content biases. Mol Biol Evol. 1992;9:678–87. 1630306 10.1093/oxfordjournals.molbev.a040752

[9] Niemeyer C, Favero CM, Kolesnikovas CKM, Bhering RCC, Brandão P, Catão-Dias JL. Two different avipoxviruses associated with pox disease in Magellanic penguins (*Spheniscus magellanicus*) along the Brazilian coast. Avian Pathol. 2013;42:546–51. 10.1080/03079457.2013.849794 24164638

