# Supplementary material for: Avian Pox in Native Captive Psittacines, Brazil, 2015
Source: Emerg Infect Dis. 2017 Jan;23(1):154–6. doi: 10.3201/eid2301.161133 (PMC5176215; doi:10.3201/eid2301.161133)
Supplement: Technical Appendix — Green-winged macaw with facial lesions, virus isolation in the chorioallantoic membrane with pocklike lesions, and the phylogenetic tree showing grouping of an isolate with Psittacinepoxvirus. [file 16-1133-Techapp-s1.pdf]

Article DOI: http://dx.doi.org/10.3201/eid2301.161133

Avian Pox in Native Captive Psittacines, Brazil, 2015

Technical Appendix

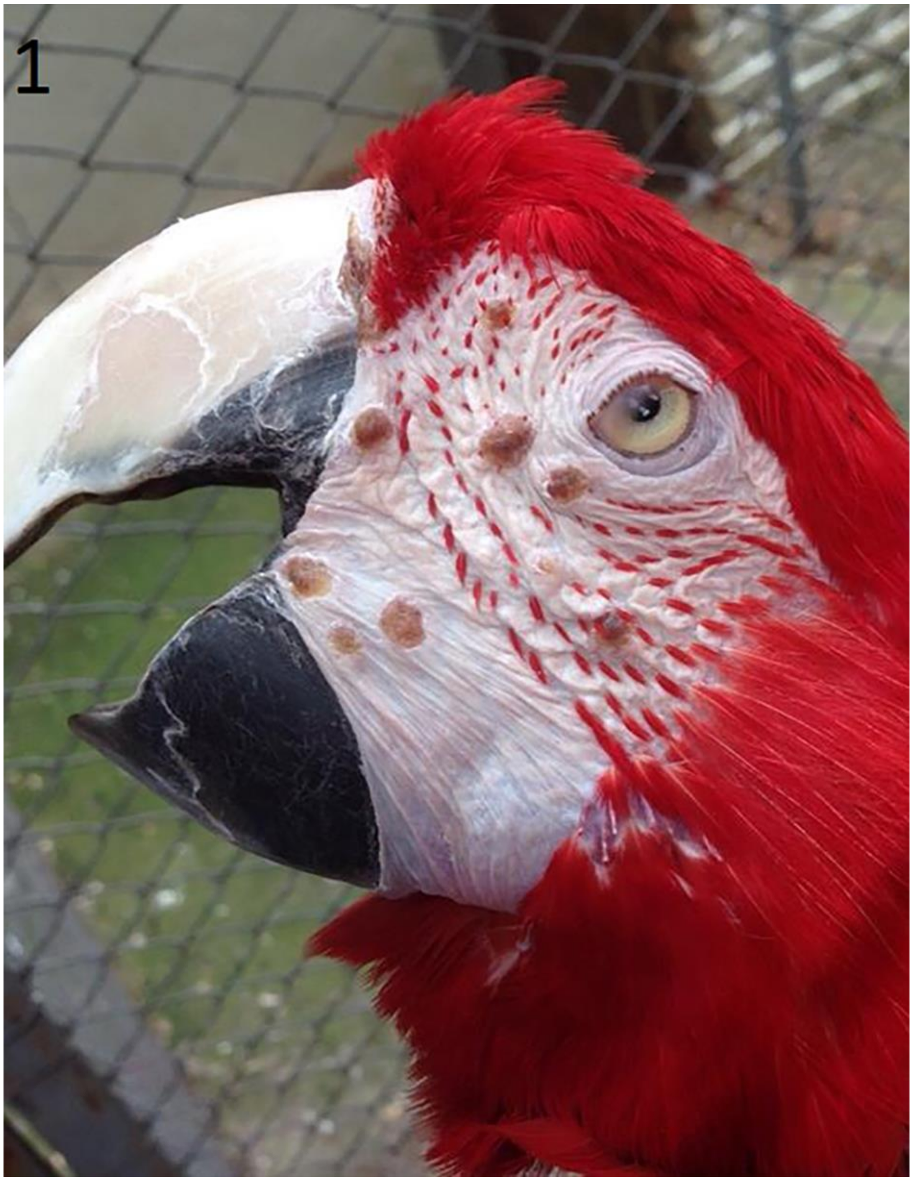

Technical Appendix Figure 1. Green-winged macaw (*Ara chloropterus*) with papular lesions on the face. This bird recovered completely.

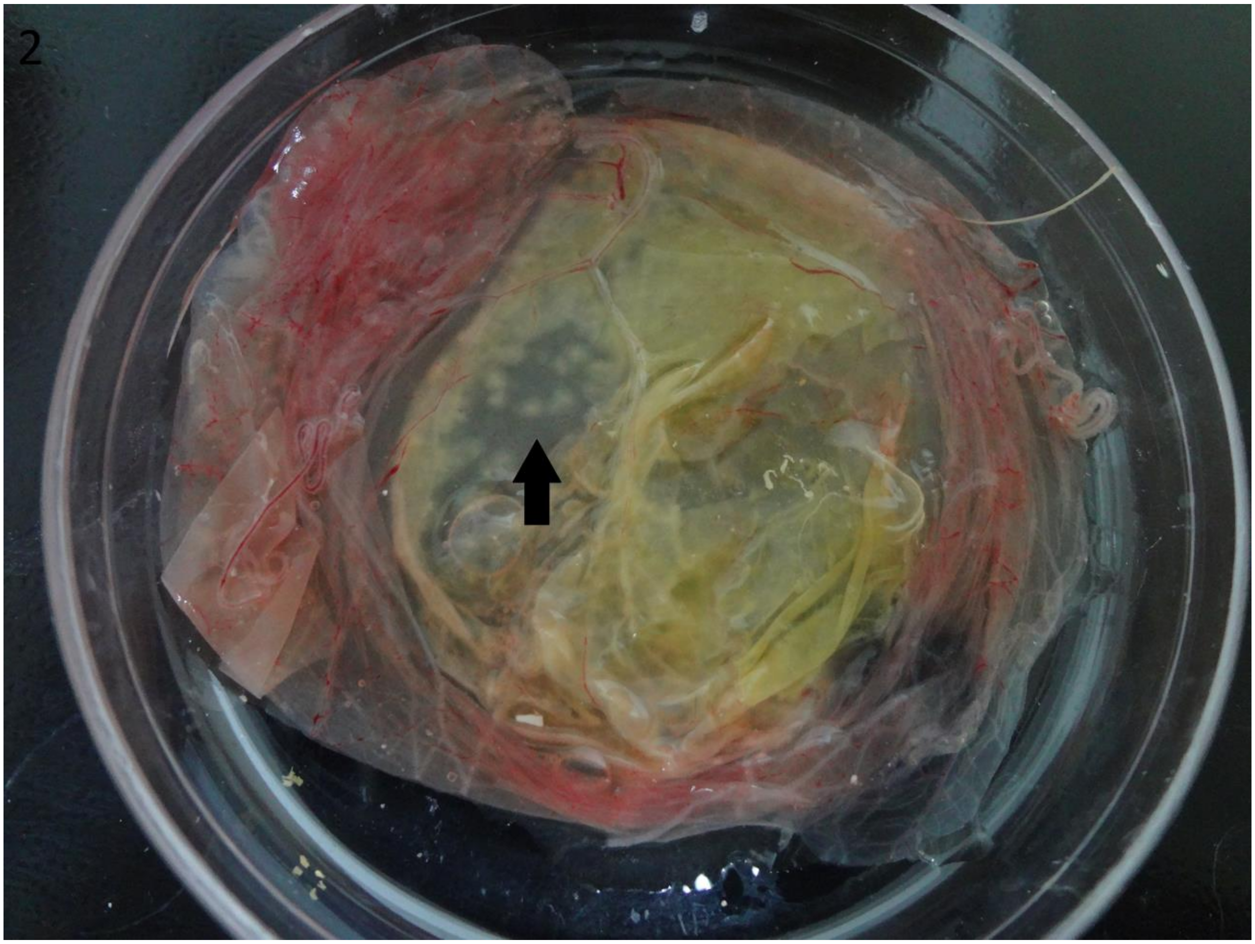

Technical Appendix Figure 2. Chorioallantoic membrane of 17-day old specific pathogen–free chicken embryo infected at 10 days of incubation with the avipoxvirus isolate of the dusky parrot (*Pionus fuscus*), showing whitish foci (arrow) and large opaque yellowish area with edema.

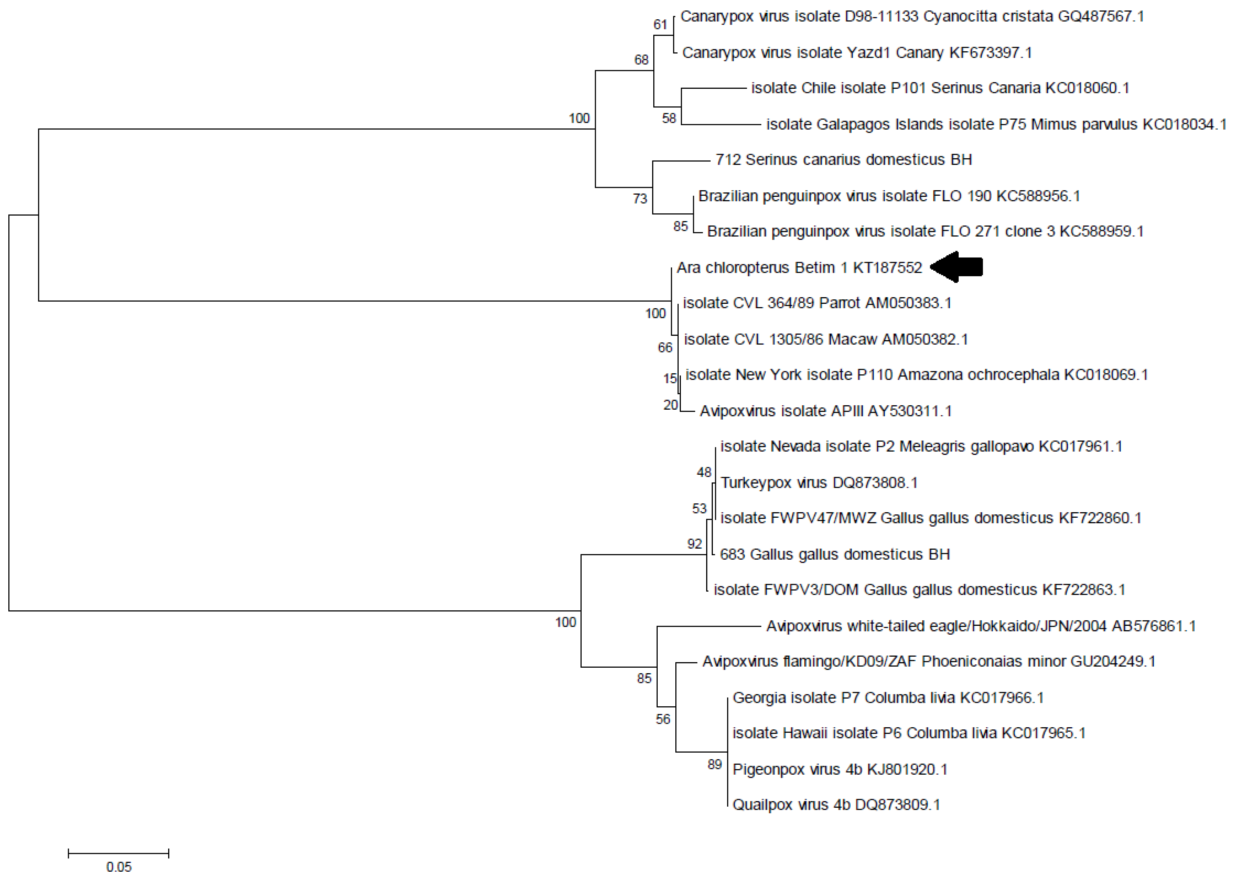

Technical Appendix Figure 3. Evolutionary relationships of avipoxvirus strains. Three phylogenetic clades were revealed, grouping *Canarypox*, *Psittacinepox*, or *Fowlpox* strains separately. The isolate *Ara chloropterus* Betim 1 (arrow) (GenBank accession no. KT187552) was grouped with *Psittacinepox* viruses, including the New York strain (KC018069.1), obtained from cutaneous lesions of the yellow-crowned amazon (*Amazona ochrocephala*), with the clinical isolates obtained in quarantine (Weybridge, UK), AM050382.1 (unspecified macaw), and AM050383.1 (unspecified parrot), and the isolate AY530311.1 from *Agapornis* (Germany). A local *Canarypox* (KX863707) isolate was grouped with the recent penguin pox strains (KC588956.1 and KC588959.1), and a local *Fowlpox* (KX863706) was grouped with chicken and turkey isolates. The evolutionary history was inferred with the neighbor-joining method (*6*). The optimal tree with the sum of branch length = 1.31754708 is shown. The percentage of replicate trees in which the associated taxa clustered together in the bootstrap test (1,000 replicates) is shown next to the branches (*7*). The tree is drawn to scale, with branch lengths in the same units as those of the evolutionary distances used to infer the phylogenetic tree. The evolutionary distances were computed by using the maximum composite likelihood method (*8*) and are in the units of the number of base substitutions per site. The analysis involved 23 nt sequences. Codon positions included were

1st+2nd+3rd+Noncoding. All positions containing gaps and missing data were eliminated; there were 470 positions in the final dataset. Evolutionary analyses were conducted in MEGA6 (*5*).
